# Increasing the Interval of Canakinumab Administration Effectively Supports the Remission of Schnitzler's Syndrome

**DOI:** 10.1155/2018/5416907

**Published:** 2018-04-11

**Authors:** Vadim R. Gorodetskiy, Svetlana O. Salugina, Evgeny S. Fedorov

**Affiliations:** V.A. Nasonova Research Institute of Rheumatology, Russian Academy of Medical Sciences, Kashirskoye Shosse 34A, Moscow 115522, Russia

## Abstract

Schnitzler's syndrome (SchS) is a rare, disabling, autoinflammatory disorder characterized by recurrent urticarial rash and monoclonal IgM gammopathy. Interleukin-1 beta (IL-1*β*) plays an important role in the pathophysiology of SchS. Only anecdotal reports demonstrate the efficiency and safety of human monoclonal anti-human IL-1*β* antibody (canakinumab) use in SchS therapy. However, there are no generally accepted recommendations concerning the scheme (or frequency) of canakinumab use for this disease. Here, we report the effective long-term treatment of SchS in a 44-year-old male with a standard canakinumab dose (150 mg) but with an increased 4-month injection interval.

## 1. Introduction

Schnitzler's syndrome (SchS) is a chronic, disabling, autoinflammatory disorder characterized by chronic urticarial rash and a monoclonal component (usually IgM) [1]. Relapsing fever, bone and muscle pain, arthralgia/arthritis, lymphadenopathy, hepato- or splenomegaly, and increased white blood cell count, erythrocyte sedimentation rate (ESR), and acute phase proteins (C-reactive protein (CRP) and serum amyloid A (SAA)) can also be observed in patients with SchS [2–5]. Adequate treatment should be provided for patients with significantly deteriorated quality of life and/or demonstrable inflammatory activity, even in the absence of significant symptoms [2]. Recently, an important role of interleukin-1 beta (IL-1*β*) was demonstrated in SchS pathophysiology [6, 7]. Canakinumab is a human monoclonal anti-human IL-1*β* antibody that binds to human IL-1*β* and neutralizes its activity by blocking its interaction with IL-1 receptors [8]. There are no generally accepted recommendations concerning the scheme of canakinumab use for SchS [7, 9–11]. We present a case of efficient and safe use of canakinumab with increased injection interval in a patient with SchS.

## 2. Case Description

**Figure 1:**
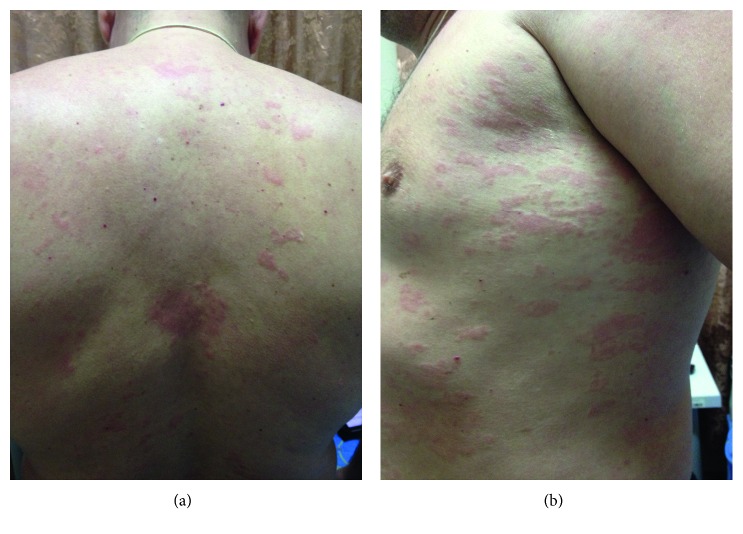
Urticarial rash on the back (a) and side of the torso (b).

A 44-year-old male was admitted to the V.A. Nasonova Research Institute of Rheumatology in November 2013 with complaints of weakness, fatigue, urticaria without evident pruritus (Figure 1), fever up to 39°C, and pain in the bilateral legs and knees. Symptoms manifested 4 years prior to presentation at the institute. The patient was diagnosed with chronic recurrent allergic urticaria. Antihistamines were ineffective; therefore, prednisone (60 mg/day) was administered, which resulted in the disappearance of clinical manifestations. After prednisone withdrawal, the symptoms recurred. At the time of admission, laboratory tests were conducted and the levels of the following were found to be high: leukocytes 23.2 × 10^9^ cells/L (normal < 9.0 cells/L), ESR 35 mm/h (normal < 10 mm/h), CRP 107 mg/L (normal < 5.0 mg/L), SAA 174 mg/L (normal < 6.4 mg/L), ferritin 717 *µ*g/L (normal < 150 *µ*g/L), and IL-6 37.8 pg/mL (normal < 5.9 pg/mL). Rheumatoid factor, antinuclear antibodies, and anti-dsDNA antibodies were not identified. Serum lactate dehydrogenase (LDH), alanine and asparagine transaminases, IL-1*β*, and complement components C3 and C4 levels were within the normal range. Ultrasonography showed minimal fluid accumulations in the pericardium and the left pleural cavity.

Based on the clinical and laboratory data, SchS syndrome was suspected. Serum protein electrophoresis and immunofixation revealed monoclonal IgM kappa secretion (5.2 g/L). Histological examination of the skin biopsy from the urticaria lesion site showed isolated lymphocytes, histiocytes, plasma cells, and segmented neutrophils in the perivascular spaces and the absence of vasculitis. Based on the presence of monoclonal IgM secretion, cytological and histological examination of bone marrow was performed. No findings to confirm Waldenstrom's macroglobulinemia were obtained. Mutations in the *NLRP3 (CIAS1)*, *MVK*, and *TNFRSF1A* genes were not identified.

The combination of urticaria rash, fever, arthralgia, polyserositis, and neutrophilic leukocytosis in the absence of specific autoantibodies suggested a differential diagnosis of adult-onset Still's disease (AOSD). The diagnosis of SchS was supported by shin bone pain, the lack of a history of pharyngitis, normal levels of transaminases and LDH, and a relatively small increase in ferritin. Paraprotein, which is always observed in patients with SchS, but not described in AOSD, was the final finding confirming the diagnosis of SchS.

Since November 2013, the patient was treated with methylprednisolone (MP) at a dose of 16 mg/day and methotrexate 20 mg/week subcutaneously resulting in symptomatic relief, but laboratory markers of inflammation remained elevated. Attempts to reduce the MP dose resulted in SchS recurrence. In March 2015, methotrexate was discontinued, and canakinumab therapy was initiated in April 2015 at a dose of 150 mg once every 8 weeks subcutaneously. The treatment resolved his symptoms and normalized the inflammatory laboratory parameters within 8 weeks. From December 2015, the interval between injections was extended to 3 months; from February 2016, the interval was extended to 4 months. Complete clinical and laboratory SchS remission persists (as of March 2018), despite the increased interval of canakinumab injections (once every 4 months). The monoclonal IgM level has remained stable. We did not observe any side effects in our patient during the observation period. By November 2015 (7 months after initiation of canakinumab therapy), methylprednisolone was discontinued.

## 3. Discussion

SchS is a rare disease; since its first description in 1972, fewer than 300 cases have been reported in the literature [2, 4]. The pathogenesis of SchS is unknown. The excellent efficacy of сanakinumab, which selectively inhibits IL-1*β*, suggests a key role of this cytokine in the pathogenesis of SchS [6, 7, 9–11].

A gain-of-function mutation in *NLRP3 (CIAS1)*, which encodes the NALP3 inflammasome leading to overproduction of IL-1, has been described in four patients with SchS [12, 13]. In our case, we did not detect mutations in *NLRP3 (CIAS1)*. Although lipopolysaccharide-induced IL-1*β* production is increased in circulating peripheral blood mononuclear cells during the symptomatic phase of SchS, serum IL-1*β* levels remain low [6, 14]. At the same time, IL-6 levels were significantly increased in patients with active SchS [6, 14]. Before сanakinumab therapy, our patient also showed an increase of serum IL-6 and normal level of IL-1*β*. The role of IL-6 in the pathogenesis of SchS has not been defined. The observation of elevated serum IL-6 is consistent with a potential role of this cytokine in active SchS [14–16]. Therefore, IL-6 might be another key inflammatory mediator in SchS. A report on the effectiveness of IL-6 inhibition in patients with SchS with no response to IL-1-inhibiting therapy seems intriguing [17]. According to the manufacturer's recommendations, the pharmacokinetic characteristics of сanakinumab allow its administration once every 8 weeks without loss of efficacy for the treatment of cryopyrin-associated periodic syndromes [8]. Currently, there are no recommendations concerning the frequency of сanakinumab administration in SchS. In most cases, сanakinumab was administered for the treatment of SchS at a dose of 150 mg every 8 weeks [7, 9–11]. Pesek and Fox were able to increase the interval of dosing to every 3 to 4 months without recurrence of symptoms in one of the two patients described [10]. In 2017, Krause et al. published the results of the first placebo-controlled study of canakinumab use in 20 patients with SchS [18]. Patients were randomly assigned to receive 150 mg doses of canakinumab or placebo subcutaneously for 7 days, followed by a 16-week open-label phase with canakinumab injections (150 mg or 300 mg) on relapse or worsening of symptoms. This study showed persistent clinical remission in six patients (four after 300 mg and two after 150 mg canakinumab injection) for 4 months. Based on our observation, injections of 150 mg сanakinumab with 3-4-month intervals were not associated with a loss of its therapeutic effect within 28 months. Despite the small number of patients reported in the literature, the increased interval between сanakinumab administrations can maintain complete clinical remission in some patients with SchS.
